# Refractory wheezing in Chinese children under 3 years of age: bronchial inflammation and airway malformation

**DOI:** 10.1186/s12887-016-0680-0

**Published:** 2016-08-27

**Authors:** Wenjing Gu, Wujun Jiang, Xinxing Zhang, Zhengrong Chen, Yongdong Yan, Li Huang, Meijuan Wang, Xuejun Shao, Shuhui Wang, Wei Ji

**Affiliations:** 1 Department of Respiration, Children’s Hospital of Soochow University, Suzhou, 215003 China; 2 Department of Clinical laboratory, Children’s Hospital of Soochow University, Suzhou, 215003 China

**Keywords:** Bronchoalveolar lavage, Neutrophil-mediated inflammation, Infant, Refractory wheezing, Airway malformation

## Abstract

**Background:**

Wheezing is a common symptom in early childhood. However, refractory wheezing is difficult to treat, and it may thus account for extensive use of medical resources. It is therefore important to improve our understanding of the pathophysiology of refractory childhood wheezing.

**Methods:**

In this descriptive study, we studied 156 children with refractory wheezing using fiberoptic bronchoscopy and bronchoalveolar lavage (BAL), and compared the results with a control group of 46 children with various pulmonary diseases but no wheezing. Etiology and cell classification were analyzed for each BAL sample.

**Results:**

Overall, 21.8 % of children with refractory wheezing had airway malformations including tracheomalacia, airway stenosis, and tracheal bronchus. The incidence of airway malformations increased to 31 % in infants under 12 months of age. A significant increase in neutrophil ratio and decrease in macrophage ratio were observed in BAL from children with refractory wheezing compared with controls. Pathogen infection led to a higher ratio of neutrophils in the wheezing group compared with controls. However, there were no significant differences in neutrophil ratios among children with various pathogen infections. Furthermore, children with refractory wheezing had a high rate of *Mycoplasma pneumoniae* infection.

**Conclusions:**

Airway malformations might play an important role in children under 3 years of age with refractory wheezing, especially in infants under 12 months of age. Neutrophil-mediated airway inflammation was characteristic of refractory wheezing in children under 3 years of age. In addition, infections such as *M. pneumoniae* may aggravate airway inflammation and affect refractory wheezing.

## Background

Wheezing in early childhood is a common but poorly characterized symptom, with a third of infants reported to experience multiple episodes of wheezing in their first 3 years [1]. Lower-airway inflammation, especially neutrophil-mediated inflammation, occurs in young children with recurrent wheezing [2, 3]. Compared with recurrent wheezing, refractory wheezing is more difficult to treat effectively and often accounts for extensive use of medical resources. It is therefore important to improve our understanding of the pathophysiology of refractory wheezing.

Congenital malformations of the lungs and airways are among several causes of irreversible airway obstruction in children who may develop various symptoms such as recurrent wheezing, cough, recurrent lower airway infection, severe dyspnea, and respiratory insufficiency [4–7]. We speculate that airway morphology may play an important role in refractory wheezing.

Fiberoptic bronchoscopy (FB) and bronchoalveolar lavage (BAL) are indispensable techniques for investigating pediatric patients with airway abnormalities and pulmonary infiltrates, and both are carried out as routine procedures in many health centers [8]. The aim of this study was to determine the cellular profile of BAL from infants with refractory wheezing in the Suzhou area, China, and to investigate airway malformations and neutrophil ratios to determine if ongoing inflammation plays a role in the development of this condition.

## Methods

### Inclusion and exclusion criteria

This descriptive, retrospective study enrolled children under 3 years of age with refractory wheezing, defined as persistent wheezing requiring at least 4 weeks of oral corticosteroid treatment after poor responses to an initial inhaled combination of corticosteroids and bronchodilators. Additionally, the symptom-free time during which children had no wheezing symptoms was no longer than 3 days in the study population. Children were excluded from the study group if they met any of the following conditions: 1) wheezing symptoms lasted for <1 month; 2) had an apparent recovery period; 3) were severely sick and unable to tolerate FB; 4) had a family history of smoking; and 5) were premature or low birthweight babies. All the participants’ parents or guardians gave their written informed consent for participation in the study. The study was approved by the ethics committee of Soochow University.

### Children with wheezing

Based on the inclusion and exclusion criteria, 156 children with wheezing who were hospitalized in the Children’s Hospital of Soochow University, China, from September 2011 to May 2014 were chosen for this study. All included patients received oral steroids. A wheezing infant was considered to be atopic if they had a strong family history of atopy (two or more direct relatives with atopy), atopic dermatitis, or a prior positive allergen test consisting of a prick test with an allergen on the skin or a radioallergosorbent test.

### Control group

There are ethical problems associated with conducting BAL cell analysis in healthy children. The control group therefore consisted of children sampled during the same period with the following conditions, in which the BAL cell analysis was relatively normal: 1) recurrent bronchitis without wheezing (*n* = 11); 2) persistent pulmonary atelectasis for at least 1 month (*n* = 3); 3) suspicion of foreign-body aspiration (*n* = 7); 4) persistent cough and final diagnosis of psychogenic cough (*n* = 12); 5) persistent laryngeal stridor with suspected laryngomalacia (*n* = 10); or 6) hemoptysis with suspected lung disease and final diagnosis of epistaxis (*n* = 3). Children in the first three groups underwent FB during remission. Children were excluded from the control group if they met any of the following criteria: 1) history of wheezing; 2) endoscopic bronchial inflammation; 3) endoscopic airway malformation; and/or 4) personal or familial history of atopy.

### Initial evaluation

Demographic, clinical, and laboratory data were collected after admission. White blood cell count, platelets, neutrophil ratio, and lymphocyte ratio were tested within 4 h after admission.

### Fiberoptic bronchoscopy

Parents/guardians were informed about the surgical risks of FB and signed consent was obtained prior to the procedure. Children were fasted for both solids and liquids for at least 6 h prior to the procedure. Intramuscular atropine sulfate 0.01–0.02 mg/kg and midazolam 0.2–0.4 mg/kg were administered as premedication. Upper and lower airway anesthesia was achieved with 2 % lidocaine. A flexible bronchoscope (Olympus CV260, Tokyo, Japan or Fujinon EB-270P, Miyoshi, Japan) was wedged into each lobe. The airways were washed three times with 1 ml/kg of prewarmed sterile 0.9 % saline solution, which was then collected by a sterile sputum-collecting pipe (Falcon 50 ml, Becton-Dickinson, Rutherford, NJ, USA). The collected BAL was used for cell counts, viral analysis, and microbiological analysis (bacteria and *Mycoplasma pneumoniae*).

### Cell counts

Differential cell counts were obtained using a modified version of Wright–Giemsa staining (Wright–Giemsa Stain, Baso Diagnostics Inc., Taiwan, China). At least 500 cells were examined for each specimen. The ratios of various cell types in total cell counts were reported.

### Microbiological analysis

BAL samples from wheezing and control children were tested for 10 types of viruses and bacteria, as well as *M. pneumoniae*. Bacteria were tested by inoculating BAL onto blood plates and examining them after incubation for 18–20 h. Bacterial growth >10^3^ colony-forming units/ml was considered significant. Viruses, including respiratory syncytial virus, adenovirus, influenza virus (A, B), and parainfluenza virus (1, 2, 3) were investigated by immunofluorescence tests using D^3^ Ultra Respiratory Virus Screening and LD Kit (Diagnostic Hybrids, OH, USA). Positive results were defined as more than five inclusion bodies detected under a fluorescence microscope. *M. pneumoniae* and viruses including rhinovirus, human metapneumovirus, and bocavirus were examined by polymerase chain reaction using a Nucleic Acid Amplification Fluorescent Reagent Kit (Ann Gene Co., Guangdong, China) according to the manufacturer’s instructions.

### Statistical analysis

Cell counts were presented as the mean ± standard deviation (SD) and as medians (25 % to 75 %). The wheezing and control groups were compared using nonparametric Mann–Whitney two-sample U-tests, and Mann–Whitney U-tests were used for unpaired data. *χ*^2^ tests were used for categorical variables. A *P* value < 0.05 was considered statistically significant.

## Results

### Demographic information

Overall, 5830 children under 3 years old were hospitalized because of wheezing between September 2011 and May 2014. Among these, 356 had refractory wheezing for at least 4 weeks (6.1 %), 54 had an apparent recovery period, 120 had a family history of smoking, and 32 were premature or low-birthweight babies. According to the exclusion criteria, 156 children with refractory wheezing were ultimately enrolled in the study (age range, 3–36 months; mean ± SD, 13.68 ± 7.08 months), of whom 121 (77.6 %) were male. The control group was aged 1–36 months (mean ± SD, 13.08 ± 10.56 months), including 35 male children (76.1 %). The age, sex ratio, and weight were similar in the wheezing and control groups (*P* > 0.05). The white blood cell count was significantly higher in the wheezing group compared with the control group (11.44 ± 4.97 vs 10.39 ± 4.28, *P* < 0.001). The neutrophil ratio was significantly higher and the lymphocyte ratio significantly lower in the wheezing group compared with the controls (36.82 ± 14.97 vs 29.00 ± 13.47 and 53.38 ± 14.38 vs 60.31 ± 13.22, respectively, *P* < 0.001). The blood platelet count was significantly higher in the wheezing group compared with the controls (394.44 ± 120.19 vs 381.48 ± 131.09, respectively, *P* < 0.001) (Table 1).

**Table 1:** Demographic data for children with refractory wheezing and controls

	Wheezing group (*n* = 156)	Control group (*n* = 46)	*P* value
Age, months	13.68 ± 7.08	13.08 ± 10.56	0.262
Sex, M/F	121/35	35/11	0.843
Weight, kg	10.56 ± 2.13	9.74 ± 3.72	0.115
Breastfeeding, Y/N	98/58	35/11	0.095
Whole blood cell analysis			
White blood cell, ×10^9^	11.44 ± 4.97	10.39 ± 4.28	<0.001
Neutrophils, %	36.82 ± 14.97	29.00 ± 13.47	<0.001
Lymphocytes, %	53.38 ± 14.38	60.31 ± 13.22	<0.001
Blood platelet, ×10^9^	394.44 ± 120.19	381.48 ± 131.09	<0.001

### Fiberoptic bronchoscopy findings

FB and BAL were well tolerated by both groups of children, and no major complications were observed. Thirty-four children (21.8 %) in the wheezing group had airway malformations including tracheomalacia (*n* = 24), airway stenosis (*n* = 6), and tracheal bronchus (*n* = 7). Among these 34 had airway malformations, 28 (82.4 %) were male and 27 (79.4 %) were under 12 months of age.

We further studied the characteristics of the airway inflammation in the wheezing group. A previous study reported that airway malformation alone could cause refractory childhood wheezing [4], and that these children might not have typical airway inflammation. We therefore analyzed the cellular contents of BAL from children with refractory wheezing without airway malformation and compared the results with the control group. We also performed a subject-to-subject comparison in the wheezing group.

The median recovery rate was 70 % (range, 45 %–80 %), with very little variation between the two compared groups. However, the recovered cell ratio showed a wide distribution in the wheezing group (Table 2).

**Table 2:** BAL cellular contents in children with refractory wheezing and controls^a^

	Refractory wheezing children without airway malformation (*n* = 122)	Control group (*n* = 46)	*P* value
Alveolar macrophages, %	60.80 ± 29.20, 67 (35.0 ~ 88.25)	87.06 ± 8.22, 90 (82.5 ~ 93.0)	<0.001
Lymphocytes, %	7.20 ± 6.46, 5 (2.0–10.0)	6.48 ± 5.52, 5 (2.0 ~ 8.0)	0.314
Neutrophils, %	31.26 ± 28.10, 25 (6.0 ~ 49.25)	6.38 ± 6.59, 4 (2.0 ~ 9.0)	<0.001
Eosinophils, %	0.75 ± 2.76, 0.0 (0.0 ~ 0.0)	0.08 ± 0.27, 0.0 (0.0 ~ 0.0)	0.040

^a^Data presented as mean ± SD, median (25th to 75th percentiles)

### Comparison between wheezing and control subjects

The neutrophil ratio was significantly higher (31.26 ± 28.10 % vs 6.38 ± 6.59 %, respectively, *P* < 0.001) and the macrophage ratio was significantly lower (60.80 ± 29.20 % vs 87.06 ± 8.22 %, respectively, *P* < 0.001) in the wheezing group compared with the control group. The eosinophil ratio was also higher in the wheezing group (0.75 ± 2.76 % vs 0.08 ± 0.27 %, respectively, *P* = 0.040). There was no significant difference in BAL lymphocyte ratio between the two groups.

### Comparisons within the wheezing group

Eighty-one of 122 (66.4 %) wheezing children were atopic and had no airway malformations. There was no significant difference in the ratio of macrophages, lymphocytes, eosinophils, or neutrophils in the BAL between atopic and nonatopic children in the wheezing group (Table 3).

**Table 3:** BAL cellular content in atopic and nonatopic children^a^

Variables	Atopic children (*n* = 81)	Nonatopic children (*n* = 41)	*P* value
Alveolar macrophages, %	56.86 ± 30.28, 61.0(33.5 ~ 85.5)	68.56 ± 25.60, 77.0 (53.5 ~ 90.0)	0.052
Lymphocytes, %	7.75 ± 7.28, 5.0 (2.0 ~ 12.0)	6.10 ± 4.30, 5.0 (2.0 ~ 10.0)	0.366
Neutrophils, %	34.89 ± 29.61, 30.0 (7.0 ~ 54.0)	24.10 ± 23.57, 15.0 (5.0 ~ 35.5)	0.062
Eosinophils, %	0.51 ± 1.28, 0.0 (0.0 ~ 0.0)	1.24 ± 4.40, 0.0 (0.0 ~ 0.0)	0.305

^a^Data presented as mean ± SD, median (25th to 75th percentiles)

In terms of pathogen analysis, we performed viral analysis, and *M. pneumoniae* and microbiological cultures for the wheezing and control groups. No pathogens were detected in the control group. Virus detection was positive in 17 (13.9 %) of the wheezing children without airway malformations, and microbiological findings were positive in 78 (63.9 %). Among them, *M. pneumoniae* showed the highest detection rate (*n* = 63, 51.6 %). In addition, other bacteria were detected in 30 wheezing children (24.6 %), including *Haemophilus influenzae* (*n* = 12), *Streptococcus pneumoniae* (*n* = 12), *Escherichia coli* (*n* = 2), *Burkholderia cepacia* (*n* = 1), *Pseudomonas aeruginosa* (*n* = 1), *Serratia marcescens* (*n* = 1), and *Enterobacter aerogenes* (*n* = 1).

**Fig. 1:**
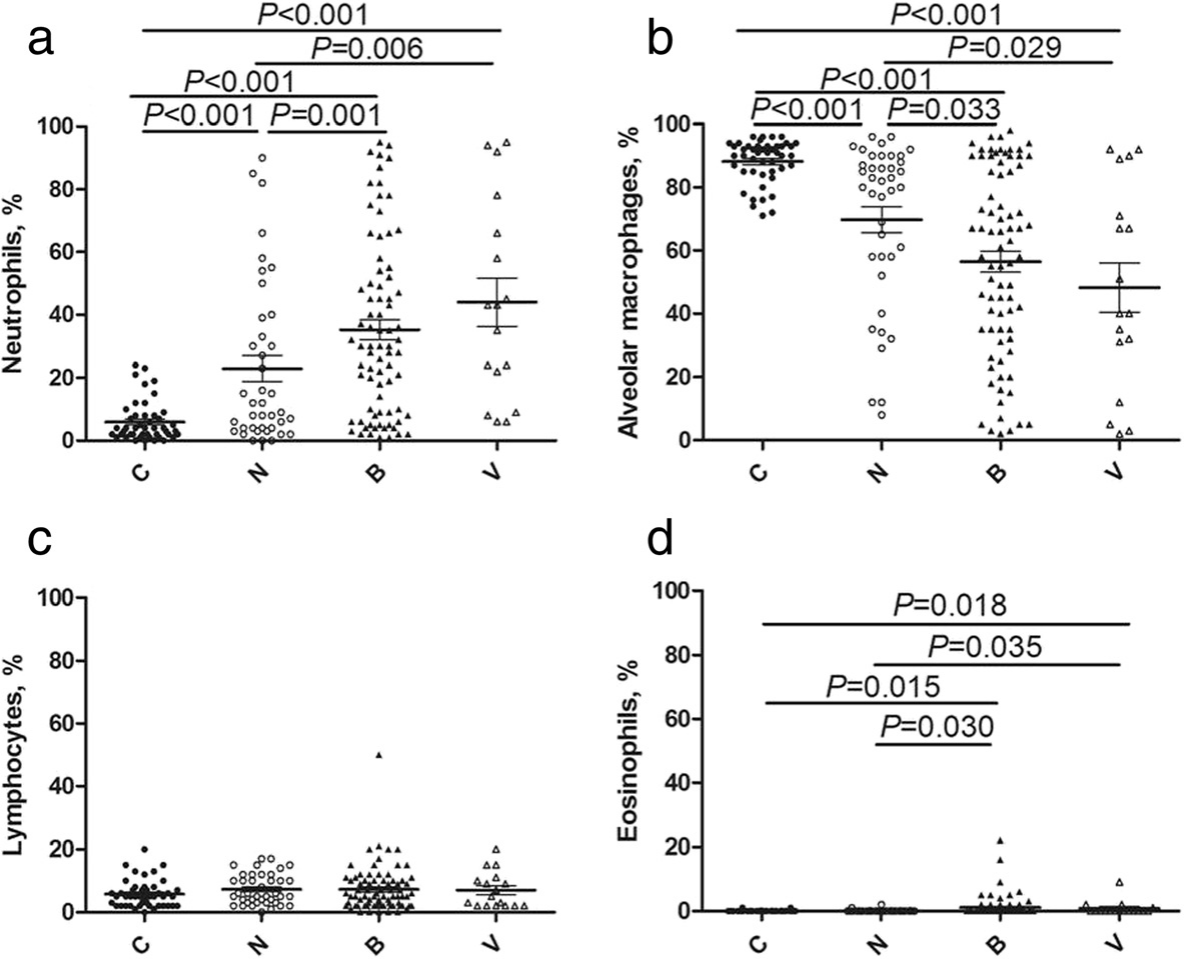
BAL cellular content. The percentages of neutrophils (**a**), alveolar macrophages (**b**), lymphocytes (**c**) and eosinophils (**d**) in the BAL in the control (C, *n* = 46), non-pathogen (N, *n* = 40), bacterial-infection (including *M. pneumoniae*) (B, *n* = 78), and virus-infection groups (V, *n* = 17). Horizontal bars indicate the median for each group of patients

We further investigated how the BAL cellular content was affected by pathogens by categorizing wheezing children into three groups: 1) non-infection group (N, *n* = 40), no pathogens detected; 2) bacterial-infection group (B, *n* = 78), bacterial infection detected (including *M. pneumoniae*); and (3) virus-infection group (V, *n* = 17), virus infection detected. We then compared the BAL cellular content between each of these groups and the control group (C, *n* = 46) (Fig. 1). The lowest neutrophil ratio was found in the control group, followed by the non-infection group, with the highest neutrophil ratios in the infection groups. There was no significant difference among the various bacterial ratios. The highest alveolar macrophage ratio was found in the control group, and the lowest in the infection groups, while the eosinophil ratio was highest in the infection groups. Of note, there was no significant difference in lymphocyte ratios among the four groups.

## Discussion

Wheezing is a common clinical symptom in infants. Its long disease course, slow recovery, and risk of relapse mean that refractory wheezing is difficult to treat, resulting in high medical-resource utilization. In our department, 6.1 % of children under 3 years of age had refractory wheezing for at least 4 weeks. Although various treatments have been used, including inhaled and systemic corticosteroids, and montelukast, they have little effect on preventing the recurrence of refractory wheezing [9]. These medications may influence eosinophilic airway inflammation but have limited impact on noneosinophilic airway inflammation. It has therefore been speculated that refractory wheezing may have a different pathophysiologic mechanism.

Airway malformation is one cause of irreversible airway obstruction in children and is accompanied by many clinical symptoms [10]. The incidence of congenital pulmonary airway malformation is one per 8300–35,000. It usually affects a single lobe and has no sex bias [11]. In this study, 21.8 % of children with refractory wheezing had airway malformations including tracheomalacia, airway stenosis, and tracheal bronchus, and 27 of 87 (31 %) wheezing children under 12 months of age had airway malformations, suggesting that airway malformations may play an important role in refractory wheezing.

A literature search [12, 13] revealed differing total BAL cell counts, though they generally fell within the range 0.5–57.1 × 10^4^/ml. Of the total BAL cells, >85 % are alveolar macrophages, <12 % are lymphocytes, <2 % are neutrophils, and <1 % are eosinophils [8, 10]. The classification of BAL cells in pathological conditions varies, and BAL analysis can provide important reference information on the nature of lung diseases, the inflammation process, and disease activity. BAL fluid analysis has recently been used globally for diagnostic and prognostic purposes, and to observe the curative effect of respiratory disease treatments. However, although it is theoretically important to study the cellular profile of BAL in healthy children, this is ethically difficult. We therefore used BAL from relatively normal children as a control, as in previous studies [3]. BAL cell analysis in our control group was comparable to that reported for healthy children [3], suggesting that the results in the control group were representative of the endotracheal cell ratio in a normal child. In the current study, we compared the BAL inflammatory cell profiles between 122 children with severe refractory wheezing and 46 control children. The BAL neutrophil ratio was significantly increased and the macrophage ratio was significantly decreased in the wheezing group compared with controls, suggesting that granulocytes contribute to airway inflammation. The mechanism of neutrophil influx and activation may be mediated by interleukin (IL)-8 secretion [14]. Studies on neutrophilic inflammation and persistent asthma by Gibson et al. revealed that sputum IL-8 levels were higher in patients with noneosinophilic asthma [15]. Neutrophils showed a close correlation with IL-8 levels [15]. Similarly, neutrophil levels were significantly higher in 83 wheezing infants compared with 10 control subjects [3]. Marguet et al. compared BAL cell profiles in children with asthma, infantile wheeze, chronic cough, and cystic fibrosis, and reported a significantly higher level of neutrophils in wheezing infants [2].

Previous studies indicated that airway virus infection and bacterial colonization can increase the risk of wheezing [16, 17]. In this study, virus detection was positive in 17 children with refractory wheezing and no airway malformation, but who had the highest bocavirus ratios. *M. pneumoniae* was positive in 63 wheezing children, and other culture findings were positive in 30 wheezing children. The *M. pneumoniae* infection rate was high in children with refractory wheezing in the current study, and previous studies also suggested that *M. pneumoniae* could cause childhood wheezing [18, 19]. However, the *M. pneumoniae* status in infants is often ignored in clinical practice. These results suggest that children with refractory wheezing should be prescribed macrolides to treat *M. pneumoniae* infection and help reduce clinical symptoms.

A high level of neutrophils in the BAL might correlate with infection. The relationships between various BAL cells counts and infection indicated a significantly higher neutrophil ratio and lower alveolar macrophage ratio in children with refractory wheezing compared with the control group, but this result was not affected by pathogen infection. However, children with a pathogenic infection had a higher neutrophil ratio and lower alveolar macrophage ratio than those without positive pathogen detection. We speculated that neutrophil-mediated chronic inflammation in the airways may also play an important role in refractory wheezing, regardless of pathogen infection, though infection may potentially aggravate the airway inflammation.

Irrespective of the pathogens, the neutrophil ratio was always higher in infected compared with uninfected children. Furthermore, there was no difference in neutrophil numbers between those infected with bacteria (including *M. pneumoniae*) or virus. Bacteria, viruses, and *M. pneumoniae* have been shown to increase the BAL neutrophil ratio. A previous study [20] found that BAL neutrophilia was associated with bacterial pulmonary infection. The neutrophil ratio has shown a tendency to increase with the occurrence of viral infection [21, 22]. This may be correlated with IL-8 and leukotriene B4, which favor the recruitment of neutrophils in airways [23–25]. *M. pneumoniae* has been studied intensively in recent years, and children with *M. pneumoniae* infection had an increased BAL cell count, which was attributed to an increase in neutrophils [26]. In contrast, other previous studies indicated that the BAL neutrophil ratio was not correlated with bacteriological results, implying that neutrophil-mediated inflammation was independent of bacterial infection [2, 3, 27] in wheezy infants.

Cellular BAL levels may be related to allergy. In our study, wheezing children were further categorized as atopic or nonatopic, and there were no significant differences in cell counts (macrophages, lymphocytes, eosinophils, and neutrophils) between the atopic and nonatopic groups. This was similar to the results of Le Bourgeois et al. [3], who observed normal BAL cell levels in atopic children. However, other studies revealed that children with atopy had higher eosinophil levels [2]. Marguet et al. [2] reported that eosinophils, which are characteristic of asthma, were rarely seen in wheezing infants. However, normal eosinophil levels do not exclude the possibility of their involvement in the physiopathology of atopic wheezing, and further studies are needed to examine the roles of activated and degranulated eosinophils. Eosinophil cationic protein (ECP) is released by activated eosinophils and is used as a marker of eosinophilic inflammation. High ECP levels have been reported in patients with active asthma and other allergic diseases [28]. The detection of ECP levels in BAL would thus be valuable [29].

## Conclusions

It is important to identify the causes associated with the pathogenesis of refractory wheezing in children. In this descriptive study, some children with refractory wheezing had airway malformations, with children under 12 months of age having a particularly high proportion of airway anomalies. Neutrophil-mediated airway inflammation might also play an important role in the pathophysiology of refractory wheezing, while infections might aggravate airway inflammation. The bacterial-detection rate (especially *M. pneumoniae*) was high among wheezing children. Further research on infants with refractory wheezing is needed to develop specific inflammatory markers.

## Abbreviations

BAL: Bronchoalveolar lavage
ECP: Eosinophil cationic protein
FB: Fiberoptic bronchoscopy
